# Evaluation of Human Ear Anatomy and Functionality by Axiomatic Design

**DOI:** 10.3390/biomimetics6020031

**Published:** 2021-05-19

**Authors:** Pratap Sriram Sundar, Chandan Chowdhury, Sagar Kamarthi

**Affiliations:** 1 Indian School of Business, Mohali 140306, India; pratap_sundar@isb.edu; 2 Indian School of Business, Gachibowli, Hyderabad 500111, India; chandan_chowdhury@isb.edu; 3 Department of Mechanical and Industrial Engineering, Northeastern University, Boston, MA 02115, USA

**Keywords:** human ear, axiomatic design, design evaluation

## Abstract

The design of the human ear is one of nature’s engineering marvels. This paper examines the merit of ear design using axiomatic design principles. The ear is the organ of both hearing and balance. A sensitive ear can hear frequencies ranging from 20 Hz to 20,000 Hz. The vestibular apparatus of the inner ear is responsible for the static and dynamic equilibrium of the human body. The ear is divided into the outer ear, middle ear, and inner ear, which play their respective functional roles in transforming sound energy into nerve impulses interpreted in the brain. The human ear has many modules, such as the pinna, auditory canal, eardrum, ossicles, eustachian tube, cochlea, semicircular canals, cochlear nerve, and vestibular nerve. Each of these modules has several subparts. This paper tabulates and maps the functional requirements (FRs) of these modules onto design parameters (DPs) that nature has already chosen. The “independence axiom” of the axiomatic design methodology is applied to analyze couplings and to evaluate if human ear design is a good design (i.e., uncoupled design) or a bad design (i.e., coupled design). The analysis revealed that the human ear is a perfect design because it is an uncoupled structure. It is not only a perfect design but also a low-cost design. The materials that are used to build the ear atom-by-atom are chiefly carbon, hydrogen, oxygen, calcium, and nitrogen. The material cost is very negligible, which amounts to only a few of dollars. After a person has deceased, materials in the human system are upcycled by nature. We consider space requirements, materials cost, and upcyclability as “constraints” in the axiomatic design. In terms of performance, the human ear design is very impressive and serves as an inspiration for designing products in industrial environments.

## 1. Introduction

The vibration of molecules in the audible frequency band creates sound, which is transmitted through air, water, or solids. The physical properties of these vibrations translate to the pitch and loudness of the sound when perceived by a human ear and other parts of the head. Nature designed the human ear to perceive sounds that are chiefly transmitted through air. Just like sight, hearing is a long-distance sense, though hearing has one advantage compared to sight: sound waves can propagate around objects. Because of this property, humans and animals can hear the sounds of an object that are not in their sight. A sensitive human ear can hear frequencies ranging from 20 Hz to 20,000 Hz. The infrasonic frequencies below 20 Hz are not detected by the human ear, but the brain senses them through the skull, jawbone, and skin, which play a vital role in the experience of music. The ultrasonic vibrations above 20,000 Hz are inaudible to the human ear, but they are employed by animals such as bats and dolphins for echolocation of objects. The human ears are most sensitive to frequencies in the 1500–4000 Hz range, which covers normal speech [1].

The ear has three regions called the outer, middle, and inner ear. The first two are solely concerned with sound transmission to the inner ear, which houses the transducer called the cochlea that converts fluid motion to action potentials. The auricle (pinna) and the ear canal, located on the outer side of the ear, collect and focus sound waves on the eardrum (tympanic membrane). The middle part of the ear, located between the eardrum and the oval window, channels sound waves from outside to inside of the ear, which houses the hammer (malleus), the anvil (incus) and the stirrup (stapes), and the eustachian tube. The inner part of the ear also includes the cochlea, the balance mechanism, the auditory nerve, and the vestibular nerve.

Governing orientation in space is the vestibular sense. Equilibrium means coordination and balance. In humans, the receptors for equilibrium constitute the vestibular apparatus, which consists of three semicircular ducts and two chambers—an anterior saccule and a posterior utricle [1].

For centuries, the workings of the ear remained mysterious. For example, scientists thought that the cochlea contains a pocket of hermetically sealed air. It was not until 1760 that the Neapolitan Domenico Cotugno declared that the entire cochlear space is filled with fluid with no room for air [2]. It took several centuries to understand the functional requirements fulfilled by the anatomical parts of the human ear. However, only in the last seventy years, did the discoveries came frequently. For example, Georg von Békésy won the Nobel prize in 1961 for his research on the function of the cochlea in the mammalian ears [3]. His research showed that different sound wave frequencies are locally dispersed prior to exciting different nerve fibers connecting the cochlea to the brain. For instance, high frequencies of a sound wave generate high vibration at the cochlea base, and the low frequencies generate more vibration at the apex. As of now, most of the functions of the components of the ear are understood, though not completely.

A survey of literature revealed that there are few research papers evaluating the design characteristics of the human ear. To the best of the authors’ knowledge, this work for the first time is evaluating the human ear design using axiomatic design framework.

In this work, the authors use axiomatic design principles [4,5,6,7,8] to assess the merit of human ear design. Axiomatic design is a well-respected approach to designing materials [9,10,11], products [12,13,14], processes [15,16], systems [17,18,19,20] and software applications [21,22,23,24,25], and for quality assurance [26,27,28,29] and decision making [30,31,32,33,34]. We apply axiomatic design-based analysis [35,36] and evaluation [37,38,39] to judge whether the human ear is a good design (i.e., uncoupled design) or a bad design (i.e., coupled design).

## 2. Axiomatic Design

Axiomatic design is a framework proposed and promoted by Nam Suh of Massachusetts Institute of Technology [4,5,6,7]. It consists of two axioms: the independent axiom and information axiom. The independent axiom expects that each functional requirement (a customer need a designer want to achieve) is satisfied by an independent design parameter in the physical domain (a physical feature that delivers the customer need). The information axiom emphasizes the selection of informationally lean and functionally reliable alternatives among the design solutions that satisfy the independent axiom. The axiomatic design framework asserts that a good design satisfies these axioms while a bad design does not. In this paper, the anatomy and functionality of the human ear is analyzed using the independent axiom. The components of the human ear are self-sufficient, reliable, and efficient. If these components were to be replaced by engineered devices delivering the equivalent functions, the latter ones would be more complex, less reliable, and less efficient. Considering this observation, it is fair to assume that the information axiom is implicitly satisfied by the ear’s design. To keep discussion of the paper simple, it focuses only on the independent axiom, which prescribes that a good design should have mutually independent functional requirements (FRs). This independence of FRs is possible when each FR is delivered by a dedicated design parameter (DP) [7]. When multiple FRs share a common DP, that DP creates coupling between the FRs. When FRs are coupled, changes in one DP results in a significant impact on two or more separate FRs. The axiomatic design introduces design matrix-based analysis to both assess and mitigate the effects of coupling. There are three types of designs: (1) uncoupled design, (2) decoupled design, and (3) coupled design. In uncoupled designs, FR-specific DPs are determined to satisfy their corresponding FRs without cross-interference. In decoupled design, DPs can be determined to satisfy FRs independently only if DPs are realized in a certain order. In coupled design, DPs cannot be determined without affecting multiple FRs, which means FRs are no longer independent. According to axiomatic design framework, the best design is a functionally uncoupled design that has minimum information content [7], and a coupled design is the least desirable one.

An interesting point of the analysis in this paper is that FRs and corresponding DPs have already been determined by nature. Scientists have discovered FRs over a long period spanning centuries of research. In this analysis, the FRs are mapped onto DPs to check for the couplings. In the sense, this work is a retrospective examination of the ear’s design through the lens of axiomatic design to discover if it is good design.

The human ear converts first pneumatic forces to mechanical forces and then to hydraulic forces, which are again converted into electrical impulses that are sent to the various parts of the central nervous system. Minute changes in the air pressure, caused by vibrations, create waves that travel through the three sections of each ear and become electrical impulses that alert the brain to a world of the sound [40]. Broadly, the ear has two major modules: The hearing module and the balancing module encased in the temporal bone, which is the strongest bone next to teeth. The hearing module converts air pressures into electrical impulses that are conveyed to the auditory cortex. The balancing module, which is also known as the vestibular system, converts body movements into electrical impulses that are conveyed to the vestibular nucleus of the pons, spinal cord, and brainstem. The cochlear and vestibular end muscles, the facial nerve, the carotid artery, and the jugular vein are all housed in the temporal bone. The following are the most significant FRs in the human ear at the highest level.

FR1 = Pick-up, amplify, transmit and process sounds.

FR2 = Balance the human body.

FR3 = Transmit sound signals to brain.

FR4 = Transmit balancing signals to the pons.

FR5 = Encompass and protect the hearing and balancing modules.

The design matrix is a diagonal matrix given below. Evidently, it is an “uncoupled design.” The proof is simple. The hearing or auditory module and the balancing or vestibular module are uncoupled. This is the reason why a deaf person can still balance his/her body, and a person with vestibular disorders can still hear and process sounds perfectly. If they are coupled, a deaf person will not be able to maintain balance, and vice versa. That could be a coupled (or bad) design. The relationship between FRs and DPs can be represented by the design equation: {FR} = [A] {DP}, where [FR] is *n* × 1 functional requirement vector, [A] is the *n* × *n* design matrix, [DP] is *n* × 1 design parameter vector, and, *n* is the number functional requirements. For *I* = 1, 2, …, *n,* and *j* = 1, 2, …, *n*, FRs in terms of DPs are given by the following expression:

$${\mathrm{FR}}_{i}=\overset{n}{\underset{j=1}{\sum }}A_{ij}{\;\mathrm{DP}}_{j}$$

$$\left\{\begin{array}{c} {\mathrm{FR}}_{1}\\ {\mathrm{FR}}_{2}\\ {\mathrm{FR}}_{3}\\ {\mathrm{FR}}_{4}\\ {\mathrm{FR}}_{5} \end{array}\right\}=\left[\begin{array}{ccccc} 1 & 0 & 0 & 0 & 0\\ 0 & 1 & 0 & 0 & 0\\ 0 & 0 & 1 & 0 & 0\\ 0 & 0 & 0 & 1 & 0\\ 0 & 0 & 0 & 0 & 1 \end{array}\right]\left\{\begin{array}{l} {\mathrm{DP}}_{1}\;\mathrm{Hearing}\;\mathrm{module}\\ {\mathrm{DP}}_{2}\;\mathrm{Balancing}\;\mathrm{module}\\ {\mathrm{DP}}_{3}\;\mathrm{Auditory}\;\mathrm{nerve}\\ {\mathrm{DP}}_{4}\;\mathrm{Vestibular}\;\mathrm{nerve}\\ {\mathrm{DP}}_{5}\;\mathrm{Temporal}\;\mathrm{bone} \end{array}\right\}$$

## 3. Outer Ear

The outer ear or the external ear, called the auricle, is the visible flap of skin on each side of the head with the auditory canal a centimeter away. The auricle is the loop of cartilage and skin that is attached to the outside of the head. The auricle functions to collect and amplify sound. Sound is funneled through the external ear and piped into the auditory canal. The ear canal, curved in an S shape, measures about 3 cm from the tragus to the tympanic membrane [1]. The FRs of the parts of the outer ear are listed below.

FR_1_ = Funnel sound and pipe into the ear canal; create notch-filtering effect; provide sound localization in vertical plane; and facilitate thermoregulation and growth.

FR_2_ = Amplify and transmit sound from concha to ear drum.

FR_3_ = Prevent small insects entering the ear.

FR_4_ = Produce cerumen that helps trap foreign particles from entering the ear and lubricate the eardrum.

FR_5_ = Sense pain.

FR_6_ = Carry signals to the brain.

FR_7_ = House the delicate parts of the ear canal and ear drum.

$$\left\{\begin{array}{c} {\mathrm{FR}}_{1}\\ {\mathrm{FR}}_{2}\\ {\mathrm{FR}}_{3}\\ {\mathrm{FR}}_{4}\\ {\mathrm{FR}}_{5}\\ {\mathrm{FR}}_{6}\\ {\mathrm{FR}}_{7} \end{array}\right\}=\left[\begin{array}{ccccccc} 1 & 0 & 0 & 0 & 0 & 0 & 0\\ 0 & 1 & 0 & 0 & 0 & 0 & 0\\ 0 & 0 & 1 & 0 & 0 & 0 & 0\\ 0 & 0 & 0 & 1 & 0 & 0 & 0\\ 0 & 0 & 0 & 0 & 1 & 0 & 0\\ 0 & 0 & 0 & 0 & 0 & 1 & 0\\ 0 & 0 & 0 & 0 & 0 & 0 & 1 \end{array}\right]\left\{\begin{array}{l} {\mathrm{DP}}_{1}\;\mathrm{Auricle}\\ {\mathrm{DP}}_{2}\;\mathrm{Ear}\;\mathrm{Canal}\\ {\mathrm{DP}}_{3}\;\mathrm{Hair}\\ {\mathrm{DP}}_{4}\;\mathrm{Glands}\\ {\mathrm{DP}}_{5}\;\mathrm{Pain}\;\mathrm{sensors}\\ {\mathrm{DP}}_{6}\;\mathrm{Sensory}\;\mathrm{neurons}\\ {\mathrm{DP}}_{7}\;\mathrm{Temporal}\;\mathrm{bone} \end{array}\right\}$$

## 4. Middle Ear

Laying between the eardrum and the oval window, the middle ear transmits sound waves from the outer ear to the inner ear. The middle ear has four components: three bones, namely, the hammer (malleus), the anvil (incus) and the stirrup (stapes), and the Eustachian tube. The middle ear serves as a pre-cochlear (pre-inner-ear) amplification system to counter the impedance mismatch between air and cochlear fluid. Without the amplification function of sound waves in the middle ear, 99.9% of the waves traveling through air would be reflected when they hit the fluid of the cochlea because the fluid has a higher impedance than air as a transmission medium [41]. The tympanic membrane (TM) or eardrum is made of a viscoelastic material shaped irregularly round, about 10 mm diameter, 0.08 mm thick, 85 square mm surface area, and 55 square mm physiologically effective area. The pressure of sound waves on the eardrum is magnified 18 times (because the area of the eardrum is larger than the area of the oval window) before the waves reach the oval widow through a funnel-like channel in the middle ear [1]. The middle ear, via ossicular coupling, provides different levels of pressure gain depending on the frequency band of the waves: the gain is about 20 dB at 250 Hz–500 Hz band; the gain reaches a peak to about 26.6 dB around 1 kHz; but the gain decreases by about 8.6 dB per octave and reaches near-zero gain at 7 kHz and above. The average middle ear sound pressure gain is about 23 dB [42].

Three tiny bones conveying vibrations need a protective function. The tensor tympani muscle and the stapedius muscle are two muscles in the middle ear that provide protection. They contract in response to loud noise, inhibiting the vibrations of the malleus, incus, and stapes and reducing the transmission of sound to the inner ear. This action is known as an acoustic reflex. The tensor tympani muscle originates from the auditory tube and attaches to the handle of the malleus, pulling it medially when contracting. It is innervated by the tensor tympani nerve, a branch of the mandibular nerve. The stapedius muscle attaches to the stapes and is innervated by the facial nerve. Contraction of both muscles is primarily activated by acoustic stimulation of 70–90 dB above the threshold. Reflex contraction takes up to 25–35 ms, stiffening the ossicular chain, reducing sound transmission by 5–10 dB, primarily at frequencies below 2 kHz [41]. This protective function may fail, resulting in an acoustic trauma, if there is a sudden sound such as an explosion or a gunshot which might take less than 25 ms to reach the ossicles.

FR_1_ = Transmit sound vibrations of the air to osscicles.

FR_2_ = Transmit sound vibrations of eardrum to oval window.

FR_3_ = Hold and support osscicles.

FR_4_ = Provide acoustic reflex to malleus bone.

FR_5_ = Provide acoustic reflex to stapes bone.

FR_6_ = Receive electrical impulses from the brain through the mandibular nerve V.

FR_7_ = Receive electrical impulses from the brain.

FR_8_ = Maintain same air pressure on both sides of the ear drum.

FR_9_ = House the delicate parts of the ear canal and ear drum.

$$\left\{\begin{array}{c} {\mathrm{FR}}_{1}\\ {\mathrm{FR}}_{2}\\ {\mathrm{FR}}_{3}\\ {\mathrm{FR}}_{4}\\ {\mathrm{FR}}_{5}\\ {\mathrm{FR}}_{6}\\ {\mathrm{FR}}_{7}\\ {\mathrm{FR}}_{8}\\ {\mathrm{FR}}_{9} \end{array}\right\}=\left[\begin{array}{ccccccccc} 1 & 0 & 0 & 0 & 0 & 0 & 0 & 0 & 0\\ 0 & 1 & 0 & 0 & 0 & 0 & 0 & 0 & 0\\ 0 & 0 & 1 & 0 & 0 & 0 & 0 & 0 & 0\\ 0 & 0 & 0 & 1 & 0 & 0 & 0 & 0 & 0\\ 0 & 0 & 0 & 0 & 1 & 0 & 0 & 0 & 0\\ 0 & 0 & 0 & 0 & 0 & 1 & 0 & 0 & 0\\ 0 & 0 & 0 & 0 & 0 & 0 & 1 & 0 & 0\\ 0 & 0 & 0 & 0 & 0 & 0 & 0 & 1 & 0\\ 0 & 0 & 0 & 0 & 0 & 0 & 0 & 0 & 1 \end{array}\right]\left\{\begin{array}{l} {\mathrm{DP}}_{1}\;\mathrm{Eardrum}\\ {\mathrm{DP}}_{2}\;\mathrm{Ossicles}\\ {\mathrm{DP}}_{3}\;\mathrm{Ligaments}\;\mathrm{of}\;\mathrm{occiscles}\\ {\mathrm{DP}}_{4}\;\mathrm{Tensor}\;\mathrm{tympani}\;\mathrm{muscle}\\ {\mathrm{DP}}_{5}\;\mathrm{Stapedius}\;\mathrm{muscle}\\ {\mathrm{DP}}_{6}\;\mathrm{Tensor}\;\mathrm{tympani}\;\mathrm{nerve}\\ {\mathrm{DP}}_{7}\;\mathrm{Facial}\;\mathrm{nerve}\\ {\mathrm{DP}}_{8}\;\mathrm{Eustachian}\;\mathrm{tube}\\ {\mathrm{DP}}_{9}\;\mathrm{Temporal}\;\mathrm{bone} \end{array}\right\}$$

## 5. Inner Ear

The inner ear is an intricate structure of delicate bones, a hollow cavity located in the skull’s temporal bone, and a system of passages. It delivers two main functions: sound detection and body balance. It includes two main functional parts: the cochlea and vestibular system. The cochlea, dedicated to hearing, converts sound pressure patterns from the outer ear into electrochemical impulses sends them as input to the brain via the auditory nerve. The vestibular system is dedicated to balancing. The eighth cranial nerve innervates the inner ear of all vertebrates. The cochlea is a labyrinth of bone, cartilage, membrane, and hair cells [1]. The FRs of the cochlea are given below, but the FRs and DPs of the vestibular system are presented in the Section 7.

FR_1_ = Receive pressure vibrations from stapes.

FR_2_ = Contain perilymph fluid on the vestibular duct.

FR_3_ = Contain perilymph fluid on the tympanic duct.

FR_4_ = Provide passage from tympanic duct to vestibular duct.

FR_5_ = Relieve the pressure created by oval window.

FR_6_ = Separate vestibular duct from cochlear duct.

FR_7_ = Transfer vibrations to the organ of Corti.

FR_8_ = Regulate volume and pressure of endolymph.

FR_9_ = Accommodate and move stereocilia.

FR_10_ = Accommodate sensory cells.

FR_11_ = Carry auditory electrical messages to the brain.

FR_12_ = House and protect the saccule, ampulla and cochlea.

$$\left\{\begin{array}{c} {\mathrm{FR}}_{1}\\ {\mathrm{FR}}_{2}\\ {\mathrm{FR}}_{3}\\ {\mathrm{FR}}_{4}\\ {\mathrm{FR}}_{5}\\ {\mathrm{FR}}_{6}\\ {\mathrm{FR}}_{7}\\ {\mathrm{FR}}_{8}\\ {\mathrm{FR}}_{9}\\ {\mathrm{FR}}_{10}\\ {\mathrm{FR}}_{11}\\ {\mathrm{FR}}_{12} \end{array}\right\}=\left[\begin{array}{cccccccccccc} 1 & 0 & 0 & 0 & 0 & 0 & 0 & 0 & 0 & 0 & 0 & 0\\ 0 & 1 & 0 & 0 & 0 & 0 & 0 & 0 & 0 & 0 & 0 & 0\\ 0 & 0 & 1 & 0 & 0 & 0 & 0 & 0 & 0 & 0 & 0 & 0\\ 0 & 0 & 0 & 1 & 0 & 0 & 0 & 0 & 0 & 0 & 0 & 0\\ 0 & 0 & 0 & 0 & 1 & 0 & 0 & 0 & 0 & 0 & 0 & 0\\ 0 & 0 & 0 & 0 & 0 & 1 & 0 & 0 & 0 & 0 & 0 & 0\\ 0 & 0 & 0 & 0 & 0 & 0 & 1 & 0 & 0 & 0 & 0 & 0\\ 0 & 0 & 0 & 0 & 0 & 0 & 0 & 1 & 0 & 0 & 0 & 0\\ 0 & 0 & 0 & 0 & 0 & 0 & 0 & 0 & 1 & 0 & 0 & 0\\ 0 & 0 & 0 & 0 & 0 & 0 & 0 & 0 & 0 & 1 & 0 & 0\\ 0 & 0 & 0 & 0 & 0 & 0 & 0 & 0 & 0 & 0 & 1 & 0\\ 0 & 0 & 0 & 0 & 0 & 0 & 0 & 0 & 0 & 0 & 0 & 1 \end{array}\right]\left\{\begin{array}{l} {\mathrm{DP}}_{1}\;\mathrm{Oval}\;\mathrm{window}\\ {\mathrm{DP}}_{2}\;\mathrm{Vestibular}\;\mathrm{duct}\\ {\mathrm{DP}}_{3}\;\mathrm{Tympanic}\;\mathrm{duct}\\ {\mathrm{DP}}_{4}\;\mathrm{Helioctrema}\\ {\mathrm{DP}}_{5}\;\mathrm{Round}\;\mathrm{window}\\ {\mathrm{DP}}_{6}\;\mathrm{Reissner}’s\;\mathrm{membrane}\\ {\mathrm{DP}}_{7}\;\mathrm{Basilar}\;\mathrm{membrane}\\ {\mathrm{DP}}_{8}\;\mathrm{Endolymph}\;\mathrm{sac}\\ {\mathrm{DP}}_{9}\;\mathrm{Tectorial}\;\mathrm{membrane}\\ {\mathrm{DP}}_{10}\;\mathrm{Organ}\;\mathrm{of}\;\mathrm{Corti}\\ {\mathrm{DP}}_{11}\;\mathrm{Cochlear}\;\mathrm{nerve}\\ {\mathrm{DP}}_{12}\;\mathrm{Bony}\;\mathrm{labyrinth} \end{array}\right\}$$

## 6. Major Functions of the Hearing System

This section considers the design matrix of the whole hearing system, which includes the outer ear, middle ear, and inner ear, and parts of the central nervous system. The outer ear has many parts such as helix, antihelix, tragus, anti-tragus, concha, and notch of the ear. Each of these parts plays an important role in capturing certain frequencies and filtering out some others. The whole system provides a gain of about 25 dB in the mid-band [43]. Some sounds bounce off the pinnae convolutions in a very useful way. The tragus and anti-tragus will reflect the bounced sound with a minute time delay. Neural components of the brain use this time delay to locate the elevation of the sound. This is the sound localization with one ear [44].

The eardrum also gives some gain. The eardrum resonates in the middle frequencies maintaining high sensitivity for the range of human voice. A conical-horn model of the human eardrum provided gain at high frequencies, most notably above 1 to 2 kHz, with broader middle-ear frequency response. This finding suggests that eardrum shape plays an important role in sound transmission to the cochlea [42].

The ear is not designed to pick up bodily sounds, such as the constant rushing sound of blood through the blood vessels near the ear. Sound waves are conducted through the bones of the skull, but the ossicles do not respond to them with the same sensitivity as they respond to tympanic movements [43]. The airborne sound is not the excitement in music and dance. The excitement comes from the sound conducted through skull bones. Similarly, the low-frequency sounds generated by acts like eating and humming are conducted through the jawbone.

The “notch-of-pinna,” due to its anatomy, almost eliminates a small band of the frequency spectrum, known as the “pinna notch.” It acts on low and high-frequency sound waves differently. Like a reflector-dish, the pinna reflects low-frequency waves toward the ear canal. In contrast, it allows a portion of high-frequency waves to directly pass through the canal, and others travel through the canal with a tiny delay. This delay eliminates the frequency component whose wave period is twice the delay period through phase cancellation. To some extent, it also attenuates the frequencies adjacent to the ones eliminated by the delay. This process affects frequencies mostly around 10 kHz, though it could influence any frequencies from 6 kHz–16 kHz [44].

The asymmetric shape of the auricle (pinna) introduces delays in the path of sound to help us achieve sound localization. In addition to auricle, the tragus also aids in sound localization. It is directionally dependent, affecting sounds coming from above more than those coming from straight ahead—this helps us achieve vertical sound localization. As sound waves pass through auricles, the waves take a spectral shape allowing the ears to detect a horizontal and vertical position of the source of the sound. If anyone or both of pinnae are covered or absent, the ability to locate the source of sound is severely impaired [44].

The ear is equipped with a truly impressive selectivity. It filters out low frequencies, amplifies mid frequencies, and enhances spatial perception through direction-sensitive filtration of high frequencies. In their own way, pinna, concha, and external ear canal perform sound wave amplification. When sound waves hit the head at 45° angle, the pinna amplifies 4 kHz frequencies to a peak of 3dB. The concha can amplify 4–5 kHz wave to a maximum gain of 10 dB. The ear canal also provides a peak gain of 10 dB at 2.5 kHz. All external ear components together amplify frequencies in the range of 2 kHz to 5 kHz with the maximal gain of 20 dB at 2.5 Hz [41]. In a room crowded with people talking, the ear can suppress most of the noise and concentrate on one speaker. From a blended sound of a symphony orchestra, the ear of the conductor can single out the one instrument that is performing improperly [45].

Sound localization is an important function fulfilled by the ear pair. If the sound originates from a location straight ahead or behind the body, the sound simultaneously reaches ears. This binaural hearing helps the brain to recognize that the source of sound is right ahead or behind the body. When the source of sound is located in a direction from the left/right of the body, the sound reaches left and right ears with a time gap and intensity variation. These interaural differences in time and intensity help the brain to locate the direction of the source of the sound [41].

**Figure 1:**
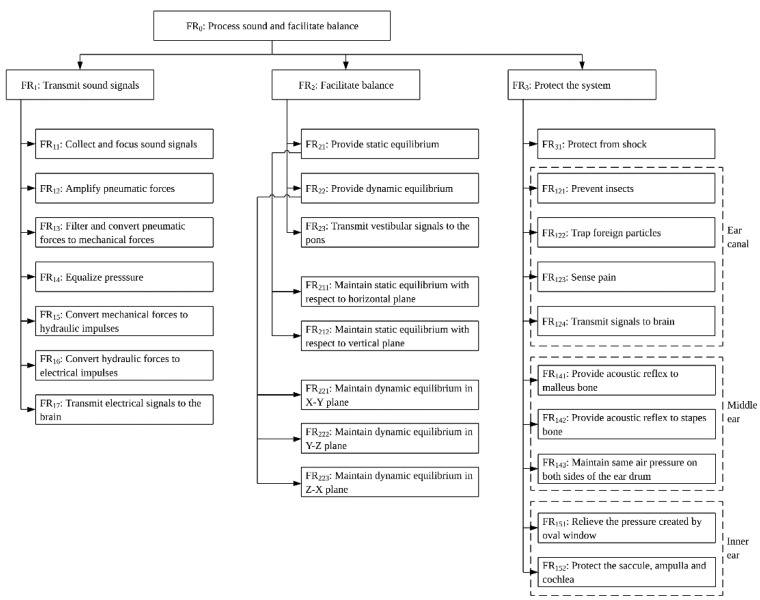
Multi-level representation of FRs.

Sound localization is achieved with either one ear (in the vertical plane) or both ears (in the horizontal plane) using the time difference and intensity of the sound waves that hit the eardrums. In addition, the human auditory system can judge if the source of sound is moving towards or away from the location of the listener. The DP that accomplishes this function is not known, but one research points out that this DP could be saccule [46]. The major FRs and the master design matrix of the hearing module are given below. Table 1 presents the master design matrix of the overall functions of hearing module. Figure 1 presents a multi-level FR-DP matrix to identify the multilevel structures of FRs and DPs.

FR_1_ = Pick-up and process sounds in audible range of 20 Hz–20,000 Hz.

FR_11_ = Maintain high sensitivity to the human voice in 1500 Hz–4000 Hz range.

FR_12_ = Ignore constant bodily sounds, whose frequencies are less than 20 Hz.

FR_13_ = Pick-up occasional bodily sounds, though their frequencies are less than 20 Hz.

FR_2_ = Amplify sounds.

FR_21_ = Amply sounds during channeling before sounds hit the eardrum.

FR_22_ = Increase pressure during conversion of pneumatic forces into mechanical forces.

FR_3_ = Filter sounds.

FR_31_ = Filter sounds at the outer ear.

FR_32_ = Filter selectively using neural components.

FR_4_ = Localize sound.

FR_41_ = Localize sound with one ear in the vertical plane.

FR_42_ = Localize sound in horizontal plane with two ears.

FR_5_ = Sense the movement of the source of sound.

**Figure 2:**
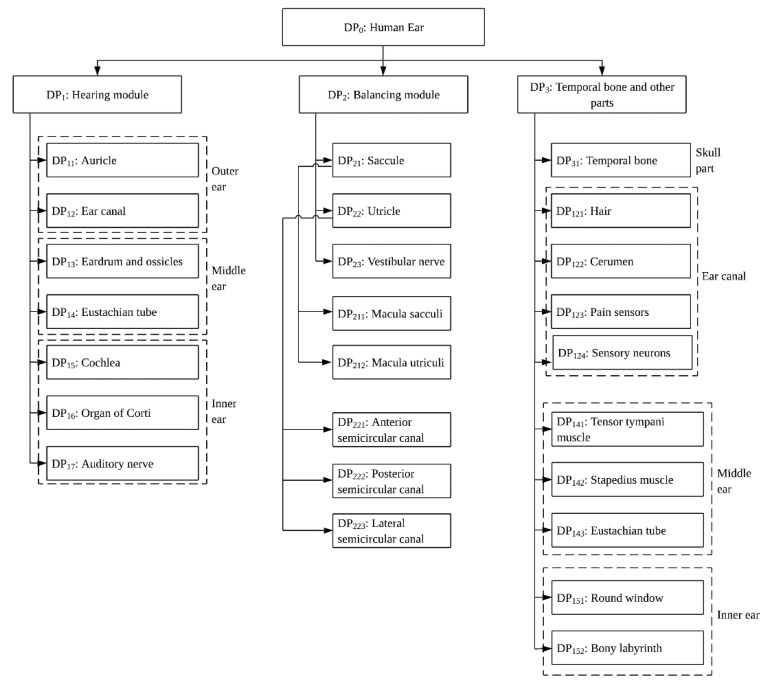
Multi-level representation of DPs.

The multi-level FRs and DPs are presented in the Figure 1 and Figure 2. Because of the space limitation the details are restricted to three levels.

## 7. Equilibrium

Certain physiological features embedded in the ear enable the sense of the body’s equilibrium. Saccule perceives the orientation of the head when the body is static. Utricle perceives linear acceleration when the body is in straight-line motion, such as when riding a car or elevator. While macula sacculi are positioned vertically on the saccule walls, macula utriculi are laid horizontally on the base of the utricle. In addition, the features shaped as semicircular ducts with about 2 × 3 mm patches of hair-like macula cells sense angular acceleration when the body is rotating or making rounds [1]. The FRs and the master design matrix showing two layers of decomposition are given below.

FR_1_ = Maintain static equilibrium.

FR_11_ = Maintain static equilibrium with respect to horizontal plane.

FR_12_ = Maintain static equilibrium with respect to vertical plane.

FR_2_ = Maintain dynamic equilibrium.

FR_21_ = Maintain dynamic equilibrium in the X–Y plane.

FR_22_ = Maintain dynamic equilibrium in the Y–Z plane.

FR_23_ = Maintain dynamic equilibrium in the Z–X plane.

Table 2 presents the master design matrix of the equilibrium function.

## 8. Constraints

Constraints (Cs) are bounds on acceptable solutions. There are two kinds of constraints: input constraints and system constraints. Input constraints are the ones imposed as part of the design specifications. System constraints are the ones imposed by the system in which the design solution must function [7]. The human ear design excels beyond expectations if it is evaluated against the space requirements, material cost, manufacturing cost, and upcyclability as constraints. The total volume occupied by the ear is about 25 cubic cm, including the outer ear. In adults, the ear canal volume is in the 0.6–1.8 mL range. The most vital part of the human ear is the cochlea, which is a spiral tube that is coiled 2.5 turns around its axis, the modiolus. It forms a cone approximately 9 mm in diameter at its base and 5 mm in height with a volume of about 0.2 milliliters. When stretched out, the spiral tube is approximately 30 mm in length [1]. It houses 3500 inner hair cells and 12,000 outer hair cells at birth. It also accommodates connections to 30,000 individual neurons. The cochlea has an abundant nerve supply of fibers taking impulses from the cochlea to the brain (afferent pathways), as well as fibers bringing impulses from the brain to the cochlea (efferent fibers). The length of each semicircular canal that signals the angular acceleration is about 15 to 22 mm, and the diameter is about 3 to 6 mm. It is difficult to achieve the miniaturization of human ear in engineered devices performing the same functions. The ear contains the smallest bone (stapes) and the smallest muscle (stapedius muscle) in the human body. Its parts are exquisitely miniature compared with most of the human-designed technological acoustic apparatus. Its functioning also incomparable to that of engineered devices. For example, at some sound frequencies, the vibrations of the eardrum are as small as one-billionth of a centimeter which is about one-tenth of the diameter of the hydrogen atom [45]. The materials that are used by nature to build the ear atom-by-atom are chiefly carbon, hydrogen, oxygen, calcium, and nitrogen. The material costs are very meager, amounting to only a few dollars. However, to calculate the manufacturing costs, one must consider the cost of energy (food) and other supplements that a human being consumes until at least 25 years of age. This cost might account for several thousand dollars appropriated to the food energy supplied to the growth, development, and maintenance of the human ear. After the death of a human body, materials in the human system are upcycled by nature. They are consumed by maggots, bacteria, and other organisms. In nature, “waste equals food” [47]. The materials are not recycled or downcycled but upcycled by nature [48].

## 9. Discussion and Conclusions

If it were an engineered device, the human ear is an excellent design that leverages sound’s physical properties: frequency (pitch), intensity (amplitude), propagation, and localization. Basically, sounds are a mix of sine waves of different frequencies and intensities, which are represented by Fourier transforms for modeling and analysis purposes. Cochlea breaks down complex sounds into their composite sine waves when it is stimulated by sound [41]. The cochlea senses the intensity of the sound measured in decibels, which quantify sound level on a logarithmic scale. This is an ingenious way of making the cochlea very compact.

The FRs satisfied by all the parts of the ear have not been completely discovered yet. For example, the outer ear has various features such as the tragus, antitragus, helix, antihelix, navicular fossa, scapha, ear-notch, concha, and lobule; the functions of some of these features are identified, but the exact function satisfied by each feature is not known. This paper presents the mapping of FRs and DPs only to a certain level of design decomposition. For example, the organ of the corti has many parts, such as tectorial membrane, outer hair cells, inner hair cells, deiters cells, each with its own FRs. So, the corti can be further decomposed to the next level, which is not presented in this paper. Similarly, further decompositions of ligaments of osscicles, components of macula sacculi, macula utriculi, semi-circular canals, and pathways of the auditory system in the central nervous system (CNS) are not presented. An analysis of the application of axiom 2 (i.e., information axiom) is also not presented in this paper.

Development of the ear begins at the third week of gestation and positions itself in full form, level with the eyes, at around the 32nd week of gestation [49]. It can grow into a bigger size (from childhood to an adult), self-repair, and self-heal. It is built inside the mother’s womb using nano-scale mechanisms: self-assembly, massive parallelism, and hierarchy. This method of manufacturing an artifact like the human ear does not use any machines, tools, operators, engineers, managers, complex scheduling systems, and quality control experts. Surprisingly, it is manufactured in a lights-out factory which is the mother’s womb.

Nature does not always create defect-free ears, perhaps because of variation in thousands of process variables that control the formation of a hearing system inside a mother’s womb. Every baby that is born does not come into this world with perfect hearing ability. About 0.3% of the babies are born every year with some form of hearing loss (mild, moderate, severe, and profound) in the United States [50]. This defect rate translates approximately to a 3-sigma quality level, with a process capability index Cpk of 1.0 or 2700 ppm. Hearing loss can also develop at any stage of life from many causes, including hereditary, birth complications, ear infections, drug side effects, and exposure to loud noise, and natural aging [51]. Most of the hearing loss defects are curable by hearing aids, cochlear implants, and other assistive devices.

Based on the analysis presented in this paper, the design of the human ear is an “uncoupled design” with only one exception: the eustachian tube, which offers microbial access to the middle ear, often leading to ear infections. The FRs can be stated as follows.

FR_1_ = Equalize pressure.

FR_2_ = Prevent microbial invasion.

$$\left\{\begin{array}{c} {\mathrm{FR}}_{1}\\ {\mathrm{FR}}_{2} \end{array}\right\}=\left[\begin{array}{cc} 1 & 1\\ 1 & 1 \end{array}\right]\left\{\begin{array}{l} {\mathrm{DP}}_{1}\;\mathrm{Open}\;\mathrm{mouth}\\ {\mathrm{DP}}_{2}\;\mathrm{Closed}\;\mathrm{mouth} \end{array}\right\}$$

The DPs are “open mouth” and “closed mouth.” When the mouth is open FR1 is satisfied. However, FR2 is violated, as the microbes will enter through the open mouth. Most of the time the eustachian tube is closed, opening only during activities such as yawning, swallowing, and chewing, or when atmospheric pressure changes rapidly (during air travel, for example) to allow air to equalize pressure in the middle ear. When the mouth is closed (which is impossible all the time), microbes may not enter, but pressure differential will remain causing symptoms such as discomfort, dizziness, or ringing in the ear. To maintain equal pressure on both sides of the eardrum, there is no other design alternative except “open mouth” in situations such as air travel. In this way, this aspect of ear design is a coupled design (a bad design). However, the human body’s immune system will attack bacteria, virus and fungus that enter through the open mouth to safeguard the functionality of the ears. When the immune system fails, medical solutions remedy the problem.

Of the FRs that have been discovered in the last seventy years (and some are yet to be discovered), when mapped onto the DPs that nature has already chosen, the human ear design seems to be an “uncoupled design,” which is the best design according to the theory of axiomatic design framework. In the field of product design and industrial design, engineers can learn a lot from nature-made design such as the human ear to mimic and emulate. There has been a lot of research in the field of biomimetics for taking models from nature and imitating the design features. The human ear has been a model for designing many audio devices, e.g., microphone design [52], middle ear prosthetics [53], hearing-aid design [54,55], cochlear implants [56,57], miniature hair bundle sensors [58], robot ear design [59,60], robot locomotion [61,62]. It would be interesting to examine how these engineered designs stack up against human ear design when examined through the lens of axiomatic design.

## Figures and Tables

**Table 1:** Master design matrix of the overall functions of hearing module.

		DP_1_			DP_2_		DP_3_		DP_4_		DP_5_ = Saccule (Probably)
		DP_11_ = Ear Canal and Ear Drum	DP_12_ = Ear (not Designed to Pick-Up the Sounds in This Range)	DP_13_ = Skull and Jaw Bones	DP_21_ = Ear Canal	DP_22_ = Ossicles	DP_31_ = Notch of the Ear	DP_32_ = Neural Components	DP_41_ = Tragus and Anti Tragus	DP_42_ = Two Ears	
FR_1_	FR_11_	1	0	0	0	0	0	0	0	0	0
	FR_12_	0	1	0	0	0	0	0	0	0	0
	FR_13_	0	0	1	0	0	0	0	0	0	0
FR_2_	FR_21_	0	0	0	1	0	0	0	0	0	0
	FR_22_	0	0	0	0	1	0	0	0	0	0
FR_3_	FR_31_	0	0	0	0	0	1	0	0	0	0
	FR_32_	0	0	0	0	0	0	1	0	0	0
FR_4_	FR_41_	0	0	0	0	0	0	0	1	0	0
	FR_42_	0	0	0	0	0	0	0	0	1	0
FR_5_	FR_5_	0	0	0	0	0	0	0	0		1

**Table 2:** Master design matrix of the equilibrium function.

		DP_1_ = Macula		DP_2_ = Semi-Circular Canals		
		DP_11_ = Macula Sacculi	DP_12_ = Macula Utriculi	DP_21_ = Anterior Semicircular Canal	DP_22_ = Posterior Semicircular Canal	DP_23_ = Lateral Semicircular Canal
FR_1_	FR_11_	1	0	0	0	0
	FR_12_	0	1	0	0	0
FR_2_	FR_21_	0	0	1	0	0
	FR_22_	0	0	0	1	0
	FR_23_	0	0	0	0	1

## Data Availability

Not applicable.
